# Room transfers and the risk of delirium incidence amongst hospitalized elderly medical patients: a case–control study

**DOI:** 10.1186/s12877-015-0070-8

**Published:** 2015-06-25

**Authors:** Amanda Goldberg, Sharon E. Straus, Jemila S. Hamid, Camilla L. Wong

**Affiliations:** Li Ka Shing Knowledge Institute, St. Michael’s Hospital, 209 Victoria Street, 7th Floor East Building, Toronto, ON M5B 1T8 Canada; Division of Geriatric Medicine, Department of Medicine, University of Toronto, Toronto, ON Canada; Department of Clinical Epidemiology and Biostatistics, McMaster University, Hamilton, ON Canada; St. Michael’s Hospital, 30 Bond Street, Toronto, ON M5B 1W8 Canada

**Keywords:** Delirium, Room transfers, Hospitalisation, Elderly medical patients

## Abstract

**Background:**

Room transfers are suspected to promote the development of delirium in hospitalized elderly patients, but no studies have systematically examined the relationship between room transfers and delirium incidence. We used a case–control study to determine if the number of room transfers per patient days is associated with an increased incidence of delirium amongst hospitalized elderly medical patients, controlling for baseline risk factors.

**Methods:**

We included patients 70 years of age or older who were admitted to the internal medicine or geriatric medicine services at St. Michael’s Hospital between October 2009 and September 2010 for more than 24 h. The cases consisted of patients who developed delirium during the first week of hospital stay. The controls consisted of patients who did not develop delirium during the first week of hospital stay. Patients with evidence of delirium at admission were excluded from the analysis. A multivariable logistic regression model was used to determine the relationship between room transfers and delirium development within the first week of hospital stay.

**Results:**

994 patients were included in the study, of which 126 developed delirium during the first week of hospital stay. Using a multivariable logistic regression model which controlled for age, gender, cognitive impairment, vision impairment, dehydration, and severe illness, room transfers per patient days were associated with delirium incidence (OR: 9.69, 95 % CI (6.20 to15.16), *P* < 0.0001).

**Conclusions:**

An increased number of room transfers per patient days is associated with an increased incidence of delirium amongst hospitalized elderly medical patients. This is an exploratory analysis and needs confirmation with larger studies.

## Background

Delirium is a clinical disorder characterized by acute and fluctuating disturbances in attention and cognition [1]. It is estimated that 14 % to 24 % of older patients are delirious upon admission to acute care hospitals, and another 6 % to 56 % of patients develop delirium while in hospital [2]. Delirium development in older patients is associated with increased morbidity and mortality, and greater healthcare costs [3–10].

Delirium results from an interaction between predisposing patient factors and precipitating hospital-related insults [2–4, 11–13]. Predisposing factors for delirium include older age, dementia, coexisting medical conditions, functional dependence, vision and hearing impairment, trauma, volume depletion, alcohol abuse, neurologic disease, and depression [2–4, 11]. Delirium precipitants in hospital include polypharmacy, psychotropic drugs, infections, sleep disruption, uncontrolled pain, metabolic derangements, use of urinary catheters and mechanical restraints, malnutrition, surgery, prolonged anaesthesia, and intensive care unit admission [2–4, 12].

The link between sensory impairment and delirium [2, 3, 11] suggests that environmental factors play a role in delirium development. Accordingly, several interventions aimed at preventing delirium included environmental modification, such as unit-wide noise reduction and placement of familiar objects, clocks, and calendars in patient rooms [14–17]. One potentially modifiable environmental factor is room transfers. Given pressures to quickly move patients out of emergency departments to hospital wards, patients are often bed-spaced to off-service units, requiring them to be moved between rooms frequently during their admission [18]. The 2010 National Institute for Health and Clinical Excellence (NICE) guidelines recommend moving patients within or between rooms be avoided unless necessary, a suggestion based on clinical experience and supported by limited data [19]. For example, McCusker and colleagues [20] demonstrated that multiple room transfers were correlated with increased delirium severity in elderly medical inpatients. However, to our knowledge, no studies have systematically examined the relationship between room transfers and delirium incidence.

We used a case–control study to determine whether room transfers are associated with an increased incidence of delirium in elderly medical patients. The secondary objectives of this study were to determine if room transfers affect delirium duration, length of stay on the internal medicine or geriatric medicine ward, and in-hospital mortality. We also sought to determine if bed-spacing or being placed in a single-bed room affect these outcomes.

## Methods

The STROBE Checklist [21] was used to report the study.

### Study population

Eligible patients were 70 years of age or older and were admitted to the internal medicine or geriatric medicine inpatient services from the emergency department at St. Michael’s Hospital in Toronto, Canada. St. Michael’s Hospital is a tertiary care academic centre with 72 internal medicine beds and 6 geriatric medicine beds. We included all patients who were admitted to these services between October 2009 and September 2010 for 24 h or longer.

### Case control selection

A validated chart abstraction technique developed by Inouye and colleagues [22] was used to identify patients with incident delirium, the primary outcome (see Appendix A). The chart based method involved a review of each patient’s emergency department records, consultation reports, medical progress notes, nursing notes, and interdisciplinary notes. We aimed to identify key terms pointing to an acute change in mental status, including delirium, confusion, mental status change, inattention, disorientation, hallucinations, agitation, inappropriate behaviour, or the presence of a constant or sitter in the patient’s room. We tried to determine, based on the notes in the chart, if the change was new and if there was any evidence of fluctuation. The abstractors coded patients as having delirium if the above-mentioned terms were present and if the patient’s presentation was not better explained by underlying dementia, structural brain abnormalities, or psychiatric illness. Three medical students participated in the chart abstraction, and the first 10 charts were abstracted by all three students to assess inter-observer agreement, determined by percent agreement. Chart abstractors could not be blinded as to when room changes occurred.

Patients who had evidence of delirium in their emergency department records were counted as prevalent cases and were excluded from the case–control study. Patients who did not have evidence of delirium in their emergency department records but who subsequently demonstrated evidence of delirium in their chart during the first week of hospital stay were considered incident cases. A one-week time frame was chosen to minimize the impact of prolonged hospitalization on delirium development. Patients who were not delirious at admission and who had no evidence of delirium in their hospital charts within the first week of stay on the internal medicine or geriatric medicine service were the controls.

### Outcome assessment: delirium duration, resolution, length of stay, mortality

The medical record pertaining to each patient’s admission was reviewed in its entirety to the date when the patient was either discharged from hospital or transferred to another service within the hospital. Delirium was considered to have resolved if the patient’s chart indicated “delirium resolved” or if the patient was consistently noted to be alert and oriented to person, place, and time. The dates on which delirium developed and resolved were recorded to calculate the duration of delirium. The length of hospital stay was defined as the number of days each patient was admitted to the internal medicine or geriatric medicine inpatient units, regardless of whether the patient was subsequently transferred to another service. The hospital’s decision support database was used to identify patients who died during their hospital stay. Only deaths that occurred while patients were admitted under the internal medicine or geriatric medicine inpatient services were used for the analysis.

### Exposures: room changes, bed-spacing, and room type

The decision support database at St. Michael’s Hospital provided a list of all the room transfers that occurred during each patient’s hospital stay and the dates on which these transfers occurred. For the purposes of this study, a transfer was defined as any move between the emergency department and the ward, between beds on different floors, between different rooms on the same floor, and between different bed spaces within the same room as this affected whether patients had exposure to a window and may have contributed to disorientation. Each patient had at least one room transfer because they were transferred from the emergency department to the inpatient ward. For the control patients, the number of room transfers that occurred within the first week of admission was recorded. For patients who developed delirium during their first week of hospitalization, the number of room transfers that occurred prior to the onset of delirium was determined.

Patients were recorded as being bed-spaced if they were admitted under the general internal medicine or geriatric medicine services, but were being cared for on a bed that belonged to a different service within the first week of hospital stay. A physical survey of the hospital wards was conducted by an investigator to identify whether rooms had one or multiple beds. Patients were recorded as being in a single-room if they were admitted to a room with one bed for their first week of hospitalization.

### Statistical analysis

Descriptive analysis was used to determine the baseline and clinical characteristics of the subjects in the study. The number of room changes before the onset of delirium was considered the primary outcome. For controls, the number of room changes during the patient’s hospital stay, up to the maximum of a week was considered, which leads to different time intervals both within the groups (within cases and controls) as well as between the groups. To allow comparability, we calculated the number of room changes per patient days (which was our primary outcome) by dividing the number of room changes by the number of days for which the number of room changes correspond to. We also compared mean number of room changes in a week as well as mean number of room changes for the entire length of stay. A comparison between case and control was performed using the *χ*^2^ test for categorical variables, Student’s *t* test for normally distributed continuous variables, and the Mann–Whitney *U* test for non-normally distributed variables. Logistic regression was conducted to examine the relationship between room transfers per patient days and incident delirium development during the first week of hospital stay, controlling for other confounders including age, gender, cognitive impairment, disease severity, vision impairment, and dehydration. To account for the difference in hospital length of stay (LOS) among cases and controls, a stratified logistic regression was performed where the LOS before delirium for cases was matched with LOS for controls. We investigated heterogeneity among the results and provided pooled estimate using meta-analytic approaches, and the results were compared with that of multiple logistic regression. Similar logistic regression was conducted to assess the relationship between bedspacing and delirium incidence and the relationship between room type and delirium incidence. All analyses were performed using SAS 9.1 software (SAS Institute Inc., Cary, NC, USA), and a p value <0.05 was considered to be statistically significant.

### Baseline risk factors

In our multivariable logistic regression, we controlled for the four baseline risk factors that were included in the Inouye prediction rule, which assessed the baseline risk factors that best predicted delirium incidence amongst a cohort of hospitalized elderly medical patients [11]. These factors include vision impairment, severe illness, cognitive impairment, and an increased blood urea nitrogen to creatinine ratio [11]. Patients noted to have a corrected visual acuity of less than 20/70 or patients noted to be legally blind were considered to have vision impairment. Patients with an APACHE II [23] score at admission of greater than 16 were considered to have severe illness. Patients with a diagnosis of dementia noted in their medical chart, or patients noted to have a Mini-Mental Status Examination score of less than 24/30 prior to admission were considered to have cognitive impairment. Finally, patients with a blood urea nitrogen (measured in mmol/L) to creatinine (measured in micromols/L) ratio of 0.1 or greater were classified as having dehydration. All of the laboratory values used to calculate the APACHE II score were abstracted from chart records obtained from patients’ first 24 h of admission. We also controlled for age and gender in our regression model.

### Ethical considerations

This study was approved by the St. Michael’s Hospital Research Ethics Board (REB# 10–377).

## Results

1386 patients were potentially eligible for the study. Among these patients, 392 (28.3 %) had evidence of delirium in their emergency department record and were excluded from the case–control study. Of the remaining 994 patients, 126 patients (12.7 %) exhibited evidence of new-onset delirium within the first week of hospitalization. These case patients were compared to the 868 control patients, and the baseline demographic characteristics of the study participants are displayed in Table 1. The admission diagnoses of the case and control patients are presented in Table 2. Patients who developed incident delirium were slightly less likely to have transient ischemic attack or stroke listed as their admission diagnosis.

**Table 1:** Summary of patient characteristics

Demographic characteristic	Patients who did not develop incident delirium (N = 868)	Patients who developed incident delirium (N = 126)	*P*-value
Age (mean, standard deviation)	81.0 (6.5)	82.7 (6.5)	0.0004
Female Gender (Percent)	498 (57.4 %)	67 (53.2 %)	0.37
Non-english speakers (Percent)	155 (17.9 %)	25 (19.9 %)	0.03
Mean length of stay	7.8 days	11.6 days	<0.0001
Mean of the number of room transfers for patients during the period of hospitalization (SD)	1.86 (1.19)	2.67 (1.59)	<0.0001
Mean number of room transfers within first week of hospital stay (SD)	1.68 (0.93)	2.21 (1.28)	<0.0001
Mean number of room transfers before the onset of delirium (SD)^*^	1.68 (0.93)	1.60 (0.90)	0.40^**^
Mean number of room transfers per patient days before delirium onset (SD)^***^	0.43	1.03	<0.0001
Number (percentage) of patients transferred between the emergency department and the ward	868 (100 %)	126 (100 %)	-
Number (percentage) of patients transferred between the medical floor and other floors in the hospital	145 (16.71 %)	12 (9.52 %)	0.04
Number (percentage) of patients transferred between different rooms on the same floor	394 (45.39 %)	56 (44.44 %)	0.84
Number (percentage) of patients transferred between different beds in the same room	51 (5.88 %)	8 (6.35 %)	0.83
Number (percentage) of patients transferred at least once (excluding ER to ward transfer)	435 (50.12 %)	92 (73.02)	<0.0001
In Total			
Within the first week of Hospitalization	396 (45.62 %)	83 (67.87 %)	<0.0001
Number (percentage) of patients transferred more than once (excluding ER to ward transfer)	171 (19.70 %)	57 (45.24 %)	<0.0001
In Total			
Within the first week of Hospitalization	135 (15.55 %)	38 (30.16 %)	<0.0001

^*^ For control groups this is the same as the number of room changes within the first week

** Note that the number of transfers after onset of delirium is not included in this comparison and hence comparison is not appropriate

^***^ Since the time periods where number of room transfers were observed are different for each patient, we adjusted it by taking the number of transfers per patient days, which is our primary outcome

**Table 2:** Summary of admission diagnoses of patients

Diagnosis	Control patients without delirium in first week (%) (N = 868)	Number of patients with incident delirium (%) (N = 126)	*P*-value (Pearson)	Odds ratios	95 % confidence intervals
Pneumonia	65 (7.49 %)	10 (7.94 %)	0.86	1.07	0.53 to 2.13
Congestive heart failure	64 (7.37 %)	12 (9.52 %)	0.40	1.32	0.69 to 2.53
Chronic obstructive pulmonary disease exacerbation	35 (4.03 %)	4 (3.17 %)	0.64	0.78	0.27 to 2.23
Gastrointestinal bleed	80 (9.22 %)	6 (4.76 %)	0.097	0.49	0.21 to 1.15
Other gastrointestinal disease	56 (6.45 %)	6 (4.76 %)	0.46	0.73	0.31 to 1.72
Urinary tract infection	37 (4.26 %)	7 (5.56 %)	0.51	1.32	0.58 to 3.03
Cancer	46 (5.30 %)	8 (6.35 %)	0.63	1.21	0.56 to 2.63
Falls/Functional decline	69 (7.95 %)	16 (12.70 %)	0.08	1.68	0.94 to 3.01
TIA/Ischemic Stroke/Intracerebral Hemorrhage	71 (8.18 %)	4 (3.17 %)	0.05	0.37	0.13 to 1.03
Endocrine/Electrolyte/Hematologic abnormality	61 (7.03 %)	11 (8.73 %)	0.49	1.27	0.65 to 2.48
Other cardiopulmonary disease	59 (6.80 %)	11 (8.73 %)	0.43	1.31	0.67 to 2.57
Renal failure	21 (2.42 %)	2 (1.59 %)	0.58	0.65	0.15 to 2.81
Pre-syncope/Syncope	17 (1.96 %)	3 (2.38 %)	0.75	1.22	0.35 to 4.23
Musculoskeletal	39 (4.45 %)	8 (6.35 %)	0.36	1.44	0.66 to 3.16
Cellulitis	19 (2.19 %)	4 (3.17)	0.49	1.47	0.49 to 4.38
Pain/Weakness	23 (2.65 %)	2 (1.59 %)	0.48	0.59	0.14 to 2.54
Other diagnoses	106 (12.21 %)	12 (9.52 %)	0.38	0.76	0.40 to 1.42

A comparison of the four baseline risk factors for delirium found in the cases and control patients is presented in Table 3. There were no statistically significant differences in the four baseline risk factors between the case and control patients.

**Table 3:** Baseline risk factors for delirium in patient population

Risk factor	Control patients without delirium in first week (Number, %) (N = 868)	Patients who developed incident delirium in first week (Number, %) (N = 126)	*P*-value	Odds ratios	95 % confidence intervals
Vision impairment	33 (3.8 %)	2 (1.6 %)	0.21	0.41	0.09 to 1.72
Cognitive impairment	138 (15.9 %)	18 (14.3 %)	0.64	0.88	0.52 to 1.50
Dehydration	249 (28.7 %)	46 (36.5 %)	0.07	1.43	0.7 to 2.11
Severe illness	155 (17.9 %)	29 (23.0 %)	0.16	1.38	0.88 to 2.16

The first 10 charts were abstracted by all three students to assess for inter-observer agreement. There was 70 % agreement across the three data abstractors regarding the delirium status of these 10 patients. The chart abstractors discussed in detail the differences in coding prior to abstracting the remainder of the charts.

### Primary outcome

Using a multivariable logistic regression model which controlled for age, gender, cognitive impairment, vision impairment, dehydration, and severe illness, room transfers per patient days was associated with increased delirium incidence (odds ratio [OR] 9.69 95 % CI (6.20, 15.16), P < 0.0001) (Table 4).

**Table 4:** Multivariable logistic regression for examining the relationship between room change per day and delirium controlling for age, gender cognitive impairment, illness severity, visual impairment, and dehydration

Variable	*P*-value	Odds ratio	95 % CI for OR
Room changes per patient day	<0.0001	9.69	(6.20,15.16)
Age	0.002	1.05	(1.02, 1.09)
Gender (F vs. M)	0.05	0.65	(0.42,1.01)
Cognitive Impairment	0.36	0.75	(0.41,1.40)
Illness severity (0 vs. 1)	0.62	0.87	(0.51,1.50)
Vision impairment	0.07	0.22	(0.04, 1.14)
Dehydration (0 vs. 1)	0.08	0.67	(0.43,1.06)

Stratified logistic regression, where hospital length of stay (LOS) before developing delirium for cases was matched with LOS for controls, indicated that the number of room transfers within the first week of admission led to an increased incidence of delirium. There were 6 strata (subgroups) – patients who developed delirium within the first, second, third, fourth, fifth and sixth days after admission. Each of these controls within each stratum were matched with controls with the same length of stay (i.e. Group 1 consists of patients who developed delirium within the first day as cases and patients who stayed 1 day in the hospital but with no sign of delirium as controls). There were no patients who developed delirium within the 7th day and hence patients with LOS = 7 are not considered as a subgroup in our analysis. Five of the 6 subgroups considered showed the number of room transfers within the first week of admission was associated with an increased incidence of delirium, where the odds ratio (OR) ranged from 2.48 to 24.51 (Table 5), however, the results were statistically significant in 2 of the 5 subgroups only. This might be due to lack of power due to very small proportion of cases within each stratum. One sub-group (for patients who developed delirium within the 3rd day), room transfer was associated with a decrease in delirium, but the result was not statistically significant (Table 5).

**Table 5:** Subgroup analysis by date of onset of incident delirium

Hospital stay date of incident delirium development	N		OR [95 % CI]	*p*-value
	Cases	Controls		
1st day	64 (58.72 %)	45 (41.28 %)	3.69 [1.59, 8.54]	0.0023*
2^nd^ day	33 (25 %)	99 (75 %)	2.48 [0.89,6.94]	0.0828
3^rd^ day	16 (12.12 %)	116 (87.88 %)	0.11 [0.005, 2.49]	0.1650
4^th^ day	15 (11.19 %)	119 (88.91 %)	8.00 [1.25, 51.35]	0.0283*
5^th^ day	7 (7.87 %)	82 (92.13 %)	22.47 [0.57, 886.95]	0.0970
6^th^ day	5 (6.67 %)	70 (93.33 %)	24.51 [0.26, 2303.72]	0.1670

^*^ Each case is matched with a control with the same LOS

**Fig. 1:**
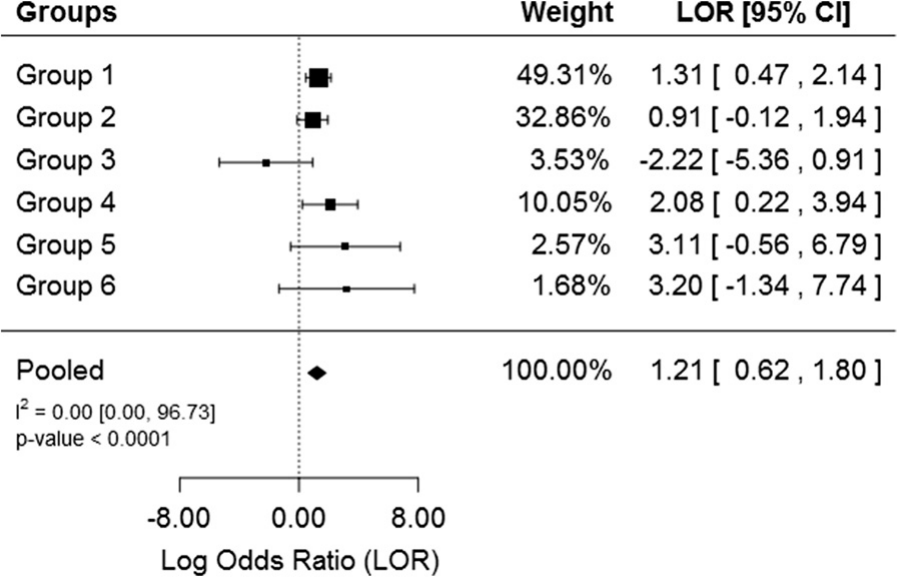
Log odds ratio of incident delirium associated with room transfers per patient days. The 6 subgroups shown represent the log odds ratio of incidence delirium within the first, second, third, fourth, fifth and sixth days after hospital admission

We explored heterogeneity among the OR estimates for the six subgroups using meta-analytic approaches and provided pooled estimates of OR (Fig. 1), as an alternative to the multiple logistic regression approach for increasing the power to detect effect of room transfer. The number of room transfers within the first week of admission led to a significant increase in incident delirium (pooled OR = 3.35, 95 % CI: [1.86, 6.05], *p*-value < 0.0001) (Fig. 1). Heterogeneity (among estimates in a log scale) was estimated to be I^2^ = 0.00 %, 95 % CI: [0.00 %, 96.73 %], indicating that the estimates were relatively homogeneous across the different subgroups (strata). However, the wide confidence interval indicates that the heterogeneity estimate was not reliable, as is the case for meta-analysis involving small number of studies.

The finding from the meta-analytic approach was in agreement with the logistic regression, where both indicated number of room transfer significantly increased incidence of delirium. However, the data sets used in the two approaches were not comparable since individuals with a LOS = 7 (all controls since none of the patients developed delirium) were not considered in the meta-analytic approach. This is because individual logistic regression could not be performed for this stratum (no cases). In order the results to be comparable, we removed patients with LOS = 7 from the analysis in the logistic regression. This resulted in an estimated OR = 5.53 (95 % CI: [3.59,8.50), *p*-value < 0.0001), which is close to the estimate obtained from the meta-analytic approach.

### Secondary outcomes

We found that an increased number of room changes per patient days during the first week of stay on the internal medicine or geriatric medicine service was associated with a decreased incidence of delirium resolution at discharge (Estimate = −1.41, P < 0.0001). Furthermore, for the 51 patients who developed incident delirium that subsequently resolved during stay under the internal medicine or geriatric medicine service, the number of room transfers per patient days was associated with a longer duration of delirium (P = 0.0003).

The number of room transfers per patient days was inversely related to length of hospital stay on the internal medicine or geriatric medicine service (Estimate = −2.57, P = 0.0003). Room type was associated with length of hospital stay on these services, such that being hospitalized in a single-bed room during the first week of hospitalization was associated with a shorter length of stay on the internal medicine or geriatric medicine services (P = 0.032).

After controlling for baseline risk factors for delirium and for the effect of room transfers on delirium incidence, there was no relationship between bed-spacing and delirium incidence within the first week of hospital stay (Table 6). There was also no relationship between room type and delirium incidence after controlling for baseline delirium risk factors and for the effect of room transfers on delirium incidence.

**Table 6:** Multivariable logistic regression for examining the relationship between bed spacing and delirium controlling for room change per day, age, gender cognitive impairment, illness severity, visual impairment, and dehydration

Variable	*P*-value	Odds ratio	95 % CI for OR
Bed-spacing (0 vs. 1)	0.18	1.37	(0.86, 2.19)
Room changes per patient day	<0.0001	9.92	(6.32,15.56)
Age	0.003	1.05	(1.02, 1.09)
Gender (F vs. M)	0.05	0.64	(0.41,1.00)
Cognitive impairment	0.33	0.74	(0.40,1.37)
Illness severity (0 vs. 1)	0.62	0.87	(0.51,1.50)
Vision impairment	0.07	0.22	(0.04, 1.17)
Dehydration (0 vs. 1)	0.09	0.67	(0.43,1.06)

There was no relationship between room transfers per patient days in the first week of hospital stay on the internal medicine or geriatric medicine services and patient mortality. Additionally, there was no relationship between bed-spacing within the first week of hospital stay on these services and patient mortality. Furthermore, no association was found between bed-spacing and length of hospital stay. There was also no relationship between room type and mortality.

Although we focused on immediate effect of room transfer (that is delirium within the first week of admission), it might be of interest to see if incident delirium was associated with outcomes such as hospital LOS and mortality. As such, we have analyzed LOS and mortality data and the results showed that LOS was higher in patients with delirium (median = 10, IQR = 9.75) than patients with no delirium in the first week of admission to the hospital (median = 5, IQR = 6). Negative binomial regression on LOS (adjusted for other baseline factors) showed that patients with incident of delirium were at a higher risk of staying in the hospital (risk ratio =1.47, 95 % CI:[1.26, 1.71], *p*-value < 0.0001). Other variables significantly associated with increased LOS were cognitive impairment, vision impairment and dehydration. Multivariable logistic regression showed that incident delirium within the first week of admission was associated with increased mortality (OR = 5.59, 95 % CI:[2.37, 13.18], *p*-value < 0.0001). However, these results should be interpreted with caution since we only considered baseline factors and factors within the first week of admission, and increase in mortality and LOS might have been due to other factors we have not considered in our study.

## Discussion

Room transfers are suspected to promote delirium development, and various multi-component delirium prevention protocols recommend minimizing room transfers [14, 24]. In this study, we found that a higher number of room transfers per patient days was strongly associated with new onset delirium within the first week of hospitalization under the internal medicine or geriatric medicine services after adjusting for baseline risk factors. Since we examined room transfers that occurred prior to the onset of delirium, it is unlikely that delirium caused the room transfers to occur.

We did not find an association between bed-spacing and delirium incidence. Likewise, we did not find an association between being in a single-person room and delirium incidence. These findings may be because room type and bed-spacing do not contribute to patient disorientation. Being placed in a single-person room was associated with a shorter length of hospital stay, possibly because some of the patients who were placed in a single room were subsequently transferred to other hospital services, thereby shortening their stay on the internal medicine ward. The increased length of stay observed in patients who developed incident delirium was likely due to the effects of delirium as delirium is known to increase length of stay [25].

Our observations are in accordance with an earlier study conducted by McCusker and colleagues [20], wherein an increased number of room changes was associated with increased delirium severity amongst hospitalized elderly patients. The finding that room changes are also associated with delirium incidence is significant because of the adverse outcomes associated with delirium development [6–10]. The delirium-provoking effects of room transfers may be related to changes in the environments and in the staff caring for patients. Prevention strategies primarily focused on patient reorientation reduced the incidence of delirium in recent studies [18, 26, 27], and should be considered in patients who undergo frequent room transfers.

### Strengths and limitations of study

The strengths of our study include a large patient population that had multiple medical illnesses and are reflective of other inpatient medicine units worldwide. Other strengths are that we limited our study to delirium incidence within the first week of hospitalization, and that we adjusted for several baseline risk factors for delirium. Our study has several limitations. Firstly, the chart abstractors could not be blinded as to how many room transfers occurred and when these transfers occurred, and this may have resulted in an overestimate of effect size, although this is unlikely. Secondly, although we controlled for the baseline risk factors in the Inouye prediction rule, residual confounding by unmeasured delirium-inducing factors likely occurred. For example, we did not evaluate precipitating factors for delirium that occurred after hospital admission [2]. Thus, increasing severity of illness may have triggered both room transfers to provide a higher level of care or an isolation room, and may have resulted in delirium, in some of the patients studied. At our institution, room transfers typically occur to make space to optimize patient flow through the emergency department. There are no policies or procedures for clinical factors outside the need to isolate patients for infections which trigger a room transfer. However, we tried to minimize the impact of prolonged hospitalization by only examining delirium incidence within the first week of hospital stay. Thirdly, our chart-based method for identifying delirium may have resulted in misclassification of some patients. Nevertheless, we used a previously validated tool, which has a reported sensitivity of 74 % and a specificity of 83 % when compared to the Confusion Assessment Method [22]. Errors may have been made in detecting hypoactive delirium, since this is often unrecognized by clinicians, [28, 29] leading to an underestimate of the incidence of delirium. Another limitation of is that we did not access patients’ primary care records to assess for baseline risk factors. This may have underestimated of the number of patients classified as having pre-existing cognitive and vision impairment. Finally, our findings can therefore only be generalized to the first week of hospitalization.

Given that room transfers represent a potentially modifiable risk factor for delirium, our findings have implications for patient care. For instance, hospital managers can mandate that transferring patients between rooms be avoided unless there is a clear rationale where the benefits outweigh the risks.

## Conclusions

In summary, although causality cannot be inferred, our results suggest that room transfers are a potentially modifiable risk factor for reducing delirium incidence amongst hospitalized elderly patients. This is an exploratory study and needs confirmation with larger, prospective studies.

## Appendix A: chart abstraction technique

The chart-based method was developed and validated by Inouye and colleagues [22]. This involved a review of each patient’s emergency department record, consultation reports, medical progress notes, nursing notes, and interdisciplinary notes. Our goal was to identify key terms indicating an acute change in mental status. We looked for the following terms: delirium, confusion, mental status change, inattention, disorientation, hallucinations, agitation, inappropriate behaviour, or the presence of a constant or sitter in the patient’s room. When these terms were identified, we tried to determine, based on the notes in the chart, if the change was new and if there was any evidence of fluctuation. The abstractors coded patients as having delirium if the above-mentioned terms were present, appeared to be acute and fluctuating, and if the patient’s presentation was not better explained by underlying dementia, structural brain abnormalities, or psychiatric illness.

In terms of the covariates, patients were recorded as having vision impairment if they were noted to be blind in one or both eyes or if they were recorded as having a corrected visual acuity of less than 20/70. If there was no mention of the patient’s visual acuity in the chart, then patients were coded as not having visual impairment. We did not have access to primary care records for the purposes of determining pre-existing vision impairment.

In terms of cognitive impairment, patients were coded as having cognitive impairment if they were noted to have dementia or cognitive impairment in the chart pertaining to the admission studied, or if they were noted to have a pre-hospitalization MMSE score of less than 24. MMSE scores were not available for the majority of patients in the study.

In terms of volume status, patients were classified as having dehydration if their blood urea nitrogen:creatinine ratio, in the emergency department, was more than 0.1. All of the patients in the study had baseline electrolyte measurements performed in the emergency department prior to admission. We used the first measurement available from the emergency department visit to assess this in cases where patients had more than one electrolyte assessment.

We used the APACHE II score to assess for illness severity. The APACHE II score is calculated using a variety of physiological and biochemical parameters. We abstracted all of these parameters from the first 24 hours of presentation. Patients with an APACHE II score of more than 16 were classified as having severe illness.

## Abbreviations

STROBE: STrengthening the Reporting of OBservational studies in Epidemiology
APACHE: Acute Physiology and Chronic Health Evaluation
